# 2762. Activity of Cefiderocol and Comparator Agents Against Difficult to Treat Resistance (DTR) Gram-negative Isolates, Collected During 2020-2022 as Part of SENTRY Antimicrobial Surveillance Program

**DOI:** 10.1093/ofid/ofad500.2373

**Published:** 2023-11-27

**Authors:** Boudewijn L DeJonge, Sean T Nguyen, Jason J Bryowsky, Christopher M Longshaw, Joshua Maher, Rodrigo E Mendes, Miki Takemura, Yoshinori Yamano

**Affiliations:** Shionogi Inc., Florham Park, New Jersey; Shionogi Inc., Florham Park, New Jersey; Shionogi Inc., Florham Park, New Jersey; Shionogi B.V., London, England, United Kingdom; JMI Laboratories, North Liberty, Iowa; JMI Laboratories, North Liberty, Iowa; Shionogi & Co., Ltd, Toyonaka, Osaka, Japan; Shionogi & Co., Ltd., Toyonaka, Osaka, Japan

## Abstract

**Background:**

Difficult to Treat Resistant (DTR) Gram-negative isolates show treatment-limiting resistance to all first-line agents; β-lactams and fluoroquinolones. Cefiderocol (CFDC) is a siderophore-conjugated cephalosporin with a unique mode of entry into bacteria, showing good activity against Gram-negative bacteria. Here, susceptibility of CFDC and comparator agents was determined against DTR isolates of Enterobacterales, *Pseudomonas aeruginosa* and *Acinetobacter baumannii-calcoaceticus* species complex (ABC).

Activity of Cefiderocol and Comparator Agents Against Difficult to Treat Resistance (DTR) Gram-negative Isolates

**Figure:**
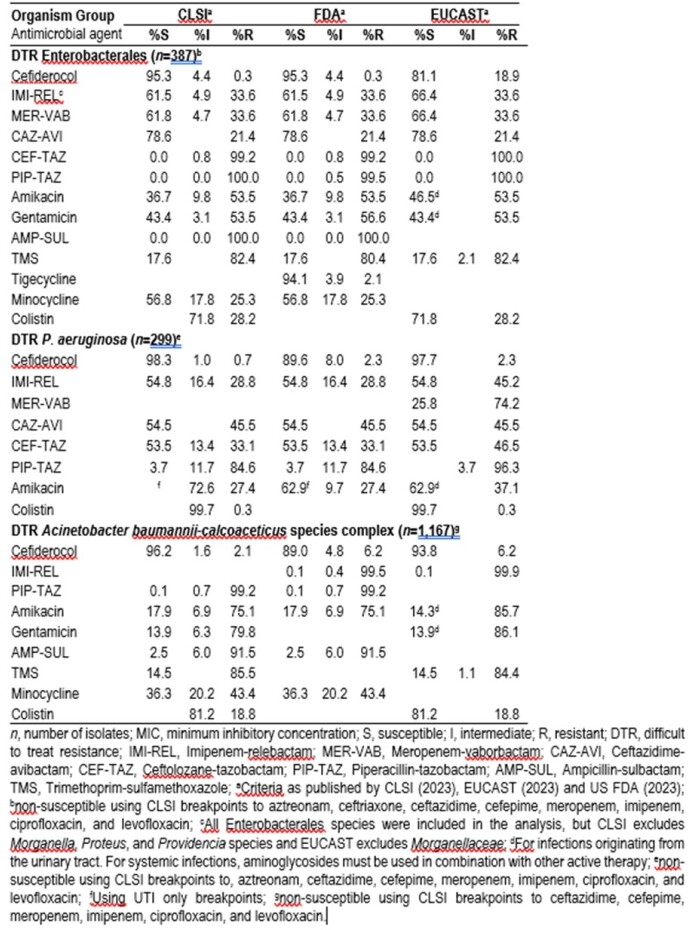

**Methods:**

Minimum inhibitory concentrations were determined according to CLSI guidelines against 24,084 Enterobacterales, 7,310 *P. aeruginosa*, and 2,479 ABC isolates, collected in 2020–2022 in Europe and the USA as part of the SENTRY program, using broth microdilution with cation-adjusted Mueller-Hinton broth (CAMHB) for comparator agents and iron-depleted CAMHB for CFDC. Susceptibility was assessed according to CLSI, EUCAST and FDA breakpoints (BPs). DTR was defined as being non-susceptible, using CLSI BPs, to fluoroquinolones and β-lactams, except for CFDC and β-lactam/β-lactamase inhibitor (BL/BLI) combinations.

**Results:**

1.6% (n=387) of the Enterobacterales isolates showed the DTR phenotype, the majority being *K. pneumoniae* (n=332; 85.8%). The isolates showed high susceptibility to CFDC (95.3% and 81.1% using CLSI/FDA or EUCAST BPs, respectively). Comparator agents, including novel BL/BLI combinations, showed susceptibility of < 80%, except for tigecycline (94.1%, FDA BPs). For *P. aeruginosa* 4.1% (n=299) showed the DTR phenotype, of which 98.3%, 97.7%, and 89.6% were susceptible to CFDC, according to CLSI, EUCAST and FDA BPs respectively. Colistin was also active (99.7% susceptible, EUCAST BPs) whereas other agents, including novel BL/BLI combinations, had susceptibility of < 65%. Amongst ABC, 47.1% (n=1167) showed the DTR phenotype, and 96.2%, 93.8%, and 89% were susceptible to CFDC according to CLSI, EUCAST and FDA BPs, respectively. Susceptibility to comparator agents was < 40%, except for colistin (81.2%, EUCAST BPs).

**Conclusion:**

DTR isolates remained susceptible to CFDC, indicative of a low degree of cross resistance with β-lactams, including BL/BLI combinations, and fluoroquinolones.

**Disclosures:**

**Boudewijn L. DeJonge, PhD**, Shionogi Inc.: Employee **Sean T. Nguyen, PharmD**, Shionogi: Employee|Shionogi, Inc: Employee **Jason J. Bryowsky, PharmD, MS**, Shionogi Inc.: Employee **Christopher M. Longshaw, PhD**, Shionogi BV: Employee **Joshua Maher, PhD**, AbbVie: Grant/Research Support|Affinity Biosensors: Grant/Research Support|AimMax Therapeutics, Inc: Grant/Research Support|Alterity Therapeutics: Grant/Research Support|Amicrobe, Inc: Grant/Research Support|Arietis Pharma: Grant/Research Support|Armata Pharmaceuticals, Inc: Grant/Research Support|Astrellas Pharma, Inc.: Grant/Research Support|Basilea Pharmaceutica AG: Grant/Research Support|Becton Dickinson And Company: Grant/Research Support|bioMerieux, Inc: Grant/Research Support|Boost Biomes: Grant/Research Support|Diamond V: Grant/Research Support|Fedora Pharmaceuticals, Inc: Grant/Research Support|Iterum Therapeutics plc: Grant/Research Support|Johnson & Johnson: Grant/Research Support|Kaleido Biosciences, Inc.: Grant/Research Support|Meiji Seika Pharma Co. Ltd.: Grant/Research Support|National Institutes of Health: Grant/Research Support|Pfizer Inc.: Grant/Research Support|Roche Holding AG: Grant/Research Support|Shionogi Inc.: Grant/Research Support|Summmit Therapeutics, Inc.: Grant/Research Support|Zoetis Inc: Grant/Research Support **Rodrigo E. Mendes, PhD**, AbbVie: Grant/Research Support|Basilea: Grant/Research Support|Cipla: Grant/Research Support|Entasis: Grant/Research Support|GSK: Grant/Research Support|Paratek: Grant/Research Support|Pfizer: Grant/Research Support|Shionogi: Grant/Research Support **Miki Takemura, n/a**, Shionogi & Co., Ltd.: Stocks/Bonds **Yoshinori Yamano, PhD**, Shionogi HQ: Employee

